# Supplementary material for: Serum Proteins Associated with Blood–Brain Barrier as Potential Biomarkers for Seizure Prediction
Source: Int J Mol Sci. 2022 Nov 25;23(23):14712. doi: 10.3390/ijms232314712 (PMC9740683; doi:10.3390/ijms232314712)
Supplement: Supplementary file 1 [file ijms-23-14712-s001.zip › ijms-1945822-supplementary/Suppl_IJMSa_corr2.pdf]

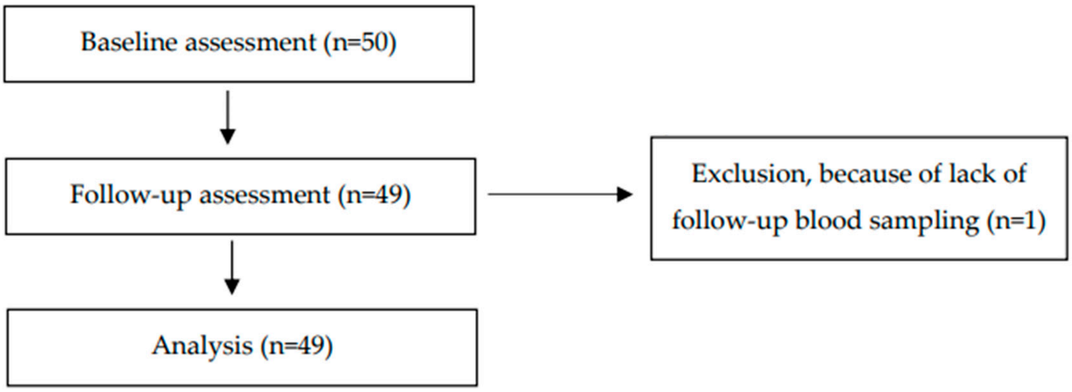

Figure S1. Study flow chart.

Table S1. Differences in the seizure count between baseline and follow-up.

	**differences in seizure count**								
	**before examination**					**after examination**			
**period [m - months. d – days]**	12 m	6 m	3 m	1 m	7 d	1 m	3 m	6 m	12 m
**mean ± SEM**	1.12 ± 1.49	(-)0.41 ± 0.73	0.41 ± 0.30	0.08 ± 0.11	(-)0.02 ± 0.03	0.00 ± 0.14	(-)0.76 ± 0.49	(-)1.31 ± 0.69	0.39 ± 1.28
**median**	0	0	0	0	0	0	0	0	0
**min-max**	(-)30 - 88	(-)25 - 34	(-)6 - 14	(-)3 - 5	(-)1 - 1	(-)6 - 5	(-)30 - 5	(-)32 - 10	(-)34 - 60

Table S2. Differences in the levels of molecules between the no-seizure and seizure group at baseline.

	**patients without seizures**			**patients with seizures**			
	**mean ± SEM**	**median**	**min-max**	**mean ± SEM**	**median**	**min-max**	**p**
**MMP-9 [ng/ml]**	605.70 ± 104.37	544.93	311.76 - 1899.46	850.18 ± 90.48	701.52	125.22 – 2555.34	0.1038
**TIMP-1 [ng/ml]**	218.93 ± 30.33	260.23	29.60 – 402.59	244.00 ± 19.61	256.50	2.69 – 464.72	0.5801
**MMP-2 [ng/ml]**	279.58 ± 32.29	243.00	155.99 - 548.59	293.89 ± 17.45	286.50	133.86 – 538.70	0.4192

**CCL-2 [pg/ml]**	311.02 ± 39.68	277.58	112.47 - 548.00	331.93 ± 25.24	315.43	118.12 – 771.43	0.6107
**P-sel [ng/ml]**	83.55 ± 20.27	38.60	6.62 - 228.47	114.25 ± 10.75	110.00	9.05 – 240.68	0.0969
**TIMP-2 [ng/ml]**	161.79 ± 11.48	156.67	112.23 – 257.58	144.88 ± 4.06	142.73	95.16 – 205.19	0.2982
**S100B [pg/ml]**	83.34 ± 40.65	41.38	0.29 – 571.13	27.30 ± 4.56	19.13	0.35 – 144.50	0.1806
**ICAM-1 [ng/ml]**	175.47 ± 15.20	161.40	103.34 – 310.27	184.48 ± 12.33	167.27	110.39 – 437.03	0.7651
**TSP-2 [ng/ml]**	32.24 ± 4.47	29.94	11.17 – 61.04	28.55 ± 3.19	21.74	8.83 – 96.59	0.3034
**MMP-9 /TIMP-1**	5.31 ± 1.99	2.08	1.33 – 27.92	8.78 ± 3.80	2.85	0.66 – 131.21	0.4585
**MMP-2 /TIMP-2**	1.74 ± 0.18	1.55	1.01 - 3.61	2.04 ± 0.11	2.05	0.83 - 3.86	0.0904

Table S3. Differences in the levels of molecules in patients without seizures at baseline and follow-up.

	**baseline**			**follow-up**			
	**mean ± SEM**	**median**	**min-max**	**mean ± SEM**	**median**	**min-max**	**p**
**MMP-9 [ng/ml]**	605.70 ± 104.37	544.93	311.76 - 1899.46	515.55 ± 82.06	444.79	68.53 – 1148.87	0.1981
**TIMP-1 [ng/ml]**	218.93 ± 30.33	260.23	29.60 – 402.59	72.04 ± 11.96	81.29	3.83 – 151.74	0.0013
**MMP-2 [ng/ml]**	279.58 ± 32.29	243.00	155.99 - 548.59	210.85 ± 20.17	201.43	97.15 – 413.73	0.1319
**CCL-2 [pg/ml]**	311.02 ± 39.68	277.58	112.47 - 548.00	430.44 ± 47.27	382.71	178.57 – 747.29	0.0047
**P-sel [ng/ml]**	83.55 ± 20.27	38.60	6.62 - 228.47	126.41 ± 29.61	69.47	19.51 – 318.63	0.3003
**TIMP-2 [ng/ml]**	161.79 ± 11.48	156.67	112.23 – 257.58	154.52 ± 7.25	158.10	106.93 – 182.97	0.6378

**S100B [pg/ml]**	83.34 ± 40.65	41.38	0.29 – 571.13	63.33 ± 28.23	27.55	0.50 – 411.25	0.7299
**ICAM-1 [ng/ml]**	175.47 ± 15.20	161.40	103.34 – 310.27	177.75 ± 18.10	166.28	112.25 – 369.73	0.9250
**TSP-2 [ng/ml]**	32.24 ± 4.47	29.94	11.17 – 61.04	25.67 ± 4.01	20.77	13.23 – 66.60	0.3305
**MMP-9 /TIMP-1**	5.31 ± 1.99	2.08	1.33 – 27.92	33.77 ± 21.01	6.61	0.45 – 299.90	0.0355
**MMP-2 /TIMP-2**	1.74 ± 0.18	1.55	1.01 - 3.61	1.37 ± 0.11	1.36	0.54 – 2.42	0.1771

Table S4. Differences in the levels of molecules in patients with seizures at baseline and follow-up.

	**baseline**			**follow-up**			
	**mean ± SEM**	**median**	**min-max**	**mean ± SEM**	**median**	**min-max**	**p**
**MMP-9 [ng/ml]**	850.18 ± 90.48	701.52	125.22 – 2555.34	423.49 ± 59.58	322.61	10.15 – 1699.80	0.0004
**TIMP-1 [ng/ml]**	244.00 ± 19.61	256.50	2.69 – 464.72	112.03 ± 9.07	117.71	9.55 – 216.35	<0.0001
**MMP-2 [ng/ml]**	293.89 ± 17.45	286.50	133.86 – 538.70	196.84 ± 17.40	211.62	41.32 – 496.85	0.0008
**CCL-2 [pg/ml]**	331.93 ± 25.24	315.43	118.12 – 771.43	436.33 ± 34.12	403.29	93.14 – 1299.29	0.0008
**P-sel [ng/ml]**	114.25 ± 10.75	110.00	9.05 – 240.68	172.17 ± 18.24	147.72	16.47 – 389.76	0.0038
**TIMP-2 [ng/ml]**	144.88 ± 4.06	142.73	95.16 – 205.19	161.51 ± 5.73	164.07	92.84 – 244.59	0.0337
**S100B [pg/ml]**	27.30 ± 4.56	19.13	0.35 – 144.50	33.94 ± 14.67	11.11	0.75 – 516.25	0.2383
**ICAM-1 [ng/ml]**	184.48 ± 12.33	167.27	110.39 – 437.03	162.72 ± 9.35	154.18	36.03 – 340.99	0.0270
**TSP-2 [ng/ml]**	28.55 ± 3.19	21.74	8.83 – 96.59	26.62 ± 1.99	22.60	11.22 – 63.50	0.6822

**MMP-9 /TIMP-1**	8.78 ± 3.80	2.85	0.66 – 131.21	6.64 ± 1.60	2.93	0.07 – 38.91	0.9347
**MMP-2 /TIMP-2**	2.04 ± 0.11	2.05	0.83 - 3.86	1.25 ± 0.11	1.29	0.29 - 3.41	0.0001

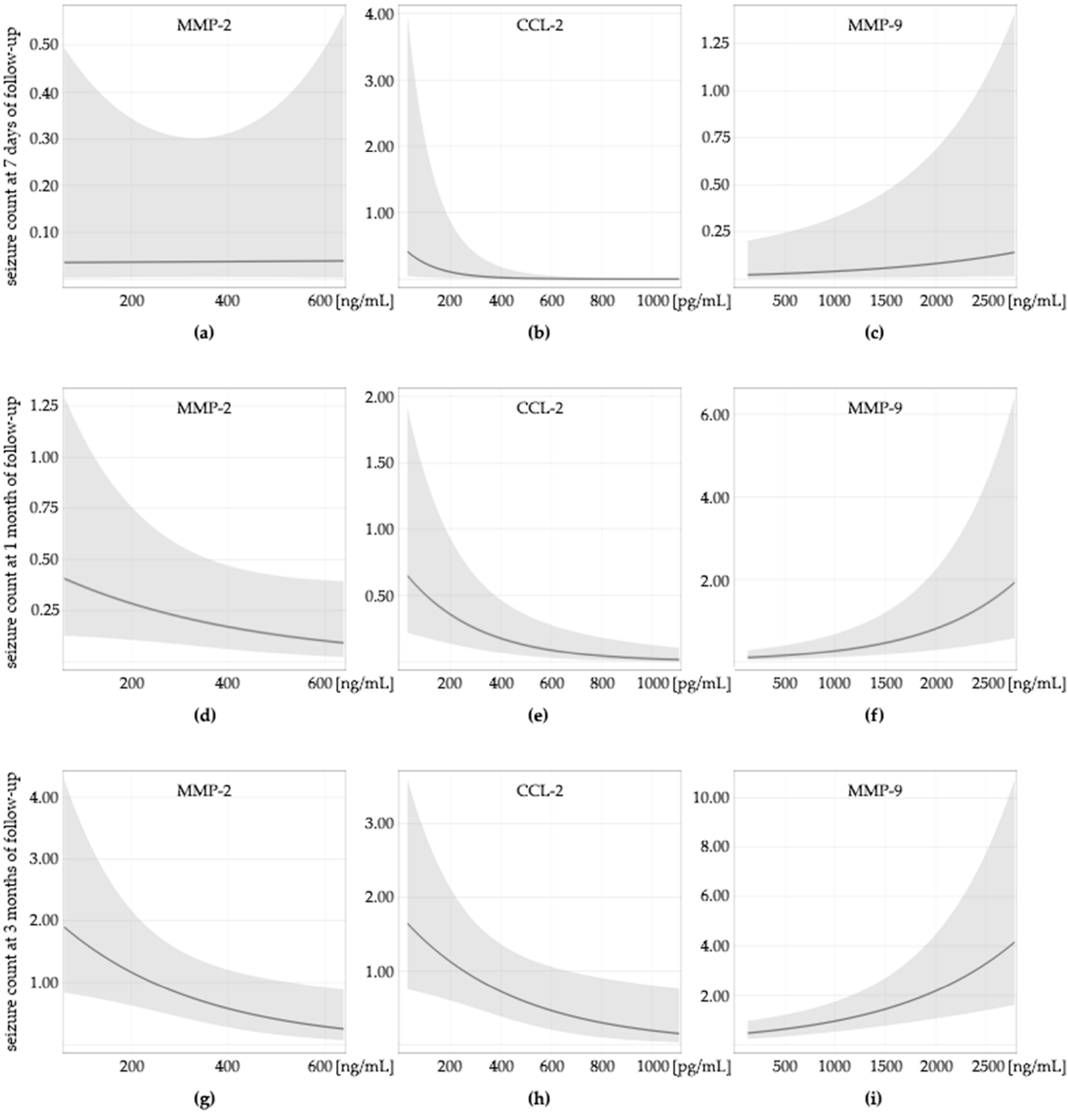

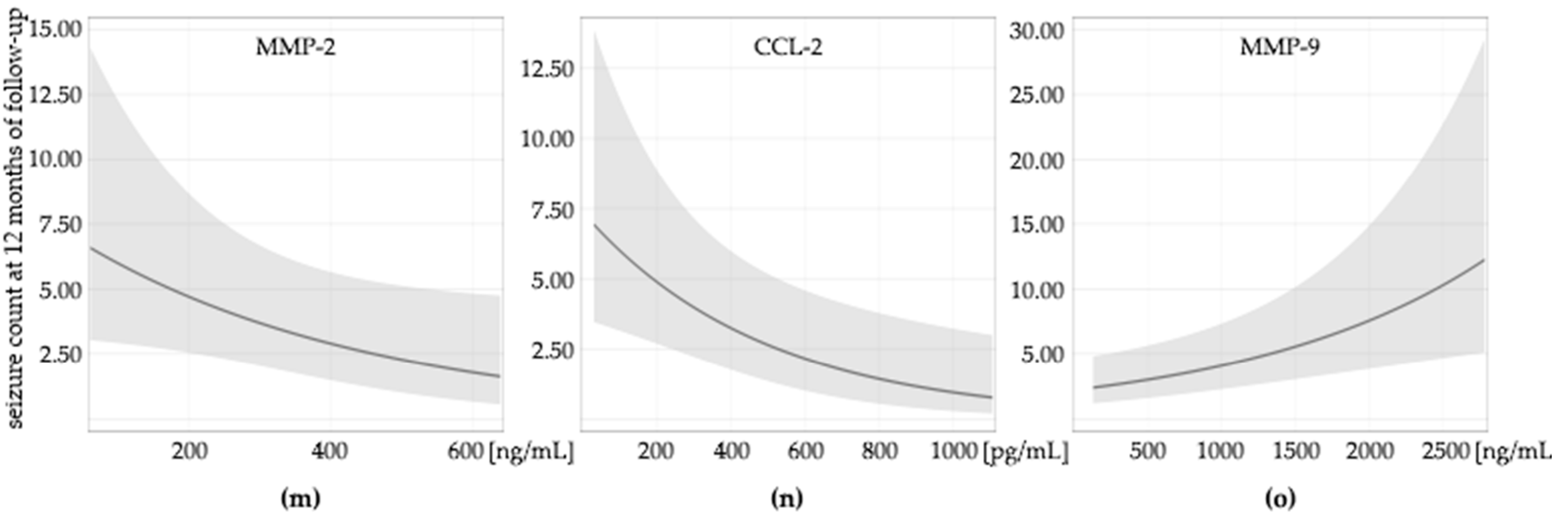

Figure S2. The influence of molecule levels on seizure count during follow-up; at 7 days: (a) MMP-2; (b) CCL-2; (c) MMP-9; 1 month (d) MMP-2; (e) CCL-2; (f) MMP-9; 3 months (g) MMP-2; (h) CCL-2; (i) MMP-9; 6 months (j) MMP-2; (k) CCL-2; (l) MMP-9; 12 months (m) MMP-2; (n) CCL-2; (o) MMP-9. GLM.
